# Exploring the Relationship Between Instagram Use and Self-Criticism, Self-Compassion, and Body Dissatisfaction in the Spanish Population: Observational Study

**DOI:** 10.2196/51957

**Published:** 2024-08-01

**Authors:** Andrea Varaona, Miguel Angel Alvarez-Mon, Irene Serrano-Garcia, Marina Díaz-Marsá, Jeffrey C L Looi, Rosa M Molina-Ruiz

**Affiliations:** 1 Department of Medicine and Medical Specialities Faculty of Medicine and Health Sciences University of Alcala Alcalá de Henares Spain; 2 CIBERSAM-ISCIII (Biomedical Research Networking Centre in Mental Health) Madrid Spain; 3 Ramón y Cajal Institute of Sanitary Research Madrid Spain; 4 Department of Psychiatry and Mental Health, Hospital Universitario Infanta Leonor Madrid Spain; 5 Department of Psychiatry and Mental Health, Hospital Clínico San Carlos Madrid Spain; 6 Academic Unit of Psychiatry and Addiction Medicine The Australian National University Medical School Canberra Australia; 7 Consortium of Australian Academic Psychiatrists for Policy, Research and Analysis Canberra Australia; 8 Fundación para la Investigación Biomédica del Hospital Clínico San Carlos Madrid Spain

**Keywords:** Instagram, self-compassion, self-esteem, self-criticism, self-worth, body dissatisfaction, dissatisfaction, satisfaction, appearance, psychological, social media, body, mental health, mental wellbeing, Spain, Spanish, Hispanic, depression, depressive, usage, correlation, association

## Abstract

**Background:**

The widespread use of online social networks, particularly among the younger demographic, has catalyzed a growing interest in exploring their influence on users’ psychological well-being. Instagram (Meta), a visually oriented platform, has garnered significant attention. Prior research has consistently indicated that Instagram usage correlates with heightened levels of perfectionism, body dissatisfaction, and diminished self-esteem. Perfectionism is closely linked to self-criticism, which entails an intense self-scrutiny and is often associated with various psychopathologies. Conversely, self-compassion has been linked to reduced levels of perfectionism and stress, while fostering greater positive affect and overall life satisfaction.

**Objective:**

This study investigates the relationship between Instagram usage (time of use and content exposure) and users’ levels of self-compassion, self-criticism, and body dissatisfaction.

**Methods:**

This study comprised 1051 adult participants aged between 18 and 50 years, either native to Spain or residing in the country for at least a decade. Each participant completed a tailored questionnaire on Instagram usage, along with abbreviated versions of the Self-Compassion Scale, the Body Shape Questionnaire, and the Depressive Experiences Questionnaire, spanning from January 23 to February 25, 2022.

**Results:**

A positive correlation was observed between daily Instagram usage and self-criticism scores. Participants of all age groups who spent over 3 hours per day on Instagram exhibited higher self-criticism scores than users who spent less than 1 hour or between 1 and 3 hours per day. Contrary to previous findings, no significant relationship was detected between Instagram usage time and levels of self-compassion or body dissatisfaction. Furthermore, content centered around physical appearance exhibited a positive correlation with self-criticism and body dissatisfaction scores. Among younger participants (aged 18-35 years), those who primarily viewed beauty or fashion content reported higher self-criticism scores than those consuming science-related content. However, this association was not significant for participants aged 35-50 years. Conversely, individuals who predominantly engaged with sports or fitness or family or friends content exhibited higher levels of body dissatisfaction than those focusing on science-related content. No significant associations were observed between self-compassion scores and daily Instagram usage or most-viewed content categories.

**Conclusions:**

The findings of this study underscore the considerable impact of Instagram usage on self-criticism and body dissatisfaction—2 variables known to influence users’ psychological well-being and be associated with various symptoms and psychological disorders.

## Introduction

The advent of social media has revolutionized how we interact and connect with others. In 2021, there were approximately 3.78 billion active users worldwide [1]. In 2022, it is estimated that Facebook (Meta) had 2.91 billion active users, followed by YouTube (Google LLC) with 2.56 billion, WhatsApp (Meta) and Instagram with 2 billion active users, and TikTok (ByteDance) with 1 billion users [2]. Social media serves as a way to stay connected with others, whether for work, educational, or social purposes [3]. The use of social networks by young adults is considered a normative experience and a new digital environment that impacts their psychological and social development [4].

According to the literature, the most visually oriented social media platforms have a greater psychological impact on users. Specifically, previous studies have examined the impact of using Instagram on self-esteem, body image satisfaction, social comparison, perfectionism, and other variables relevant to psychological well-being [5-12]. College women who used Facebook and Instagram reported a greater number of appearance-related thoughts than those who used other less visual platforms [5]. The results from a different study showed that the use of Instagram was closely related to dysmorphic concerns in men and women [7]. Moreover, in other studies, it has been observed that individuals who use Instagram for more hours per day tend to exhibit lower levels of self-esteem and psychological well-being, as well as greater dissatisfaction with their body image [10,12].

However, the association between Instagram usage and self-compassion and self-criticism has not been thoroughly studied yet. In a recent study, it was reported that users with higher levels of self-compassion used Instagram less frequently [13]. Self-criticism, besides hurting people’s moods, is also considered a vulnerability factor for the development of depression and other mental disorders, such as social anxiety, eating disorders, posttraumatic stress disorder, personality disorders, and psychotic symptoms [14-18]. The implication of self-criticism in a wide range of psychopathologies has led us to consider it as a transdiagnostic process [16,17]. Furthermore, self-criticism has been associated with self-injuries and suicidal thoughts [19-22]. On the other hand, self-compassion has been found to have multiple psychological benefits [13,23,24]. Therefore, the objective of this study was to explore the potential associations between daily Instagram usage time and the most viewed content with levels of self-compassion, self-criticism, and body dissatisfaction among users.

## Methods

### Study Design and Participants

This study uses a cross-sectional observational design, wherein participants were recruited anonymously and voluntarily. The survey was administered using Google Forms and commenced with an introductory section outlining this study’s objectives, along with obtaining informed consent regarding anonymity and voluntary participation. Participants were then prompted to provide responses regarding sociodemographic information, Instagram usage patterns, and other pertinent measures relevant to this study’s aims.

The recruitment process involved promoting the survey through one of the authors’ Instagram profiles dedicated to mental health, which boasts over 100,000 followers. Additionally, some followers shared the survey on their own Instagram stories, extending this study’s reach to a broader audience, whereas other authors shared the survey via email and WhatsApp. Data collection took place over the course of 1 month, from January 23 to February 25, 2022.

The inclusion criteria were (1) being aged between 18 and 40 years, (2) regular usage of Instagram, and (3) Spanish nationality or living in Spain for at least 10 years. Participants with a current or past diagnosis of eating disorders were excluded from this study.

### Assessment Instruments and Variables Considered

We used the following measurement instruments: (1) our own researcher-designed questionnaire collecting sociodemographic data and Instagram use variables, (2) the Self-Compassion Scale (SCS); (3) the Depressive Experiences Questionnaire (DEQ); (4) the Body Shape Questionnaire (BSQ). In the Instagram questionnaire (available in Multimedia Appendix 1), aspects such as daily usage time on Instagram, the number of followers, the number of accounts they followed, the most viewed content (science, gastronomy, traveling, family or friends, beauty or fashion, sports, fitness, or lifestyle, humor, news, or others), and the number of stories and posts each participant published daily were also assessed.

Regarding the SCS [25], we used the short version translated into Spanish and validated by Garcia-Campayo et al [26]. This self-applied scale consists of 12 items that assess self-compassion in general (total score) and 3 specific components of self-compassion: common humanity, mindfulness, and self-compassion. Although the construct of self-compassion is defined from these 3 components, the scale is divided into 6 subscales representing the positive and negative aspects of each of these 3 components: self-compassion, self-judgment, common humanity, isolation, mindfulness, and overidentification. Participants are asked to respond to the items according to how they usually act toward themselves in difficult times using a Likert-type scale ranging from 1 (“almost never”) to 5 (“almost always”). In this short Spanish version, Cronbach α is 0.85, indicating good internal consistency. As for the correlation between the items of the 26-item and 12-item versions, high correlations were observed between the different subscales: self-compassion (*r*=0.89), self-judgment (*r*=0.90), common humanity (*r*=0.81), isolation (*r*=0.81), mindfulness (*r*=0.83), and overidentification (*r*=0.90). Regarding the correlation between the total score of both versions, a high correlation was also observed (*r*≥0.92). Higher scores on this scale are considered to indicate higher levels of self-compassion [27].

Furthermore, we used the DEQ [28]. The short version was translated into Spanish and validated [29]. This questionnaire is composed of 32 items that measure 3 factors: dependence, self-criticism, and relationship. In this version, the relationship factor is grouped within the dependence factor. Although both refer to the phenomenon of separation, the dependency factor concerns a more primitive level of development and refers to feelings of helplessness, hopelessness, and fear of separation, while the relationship factor refers to feelings of loss and loneliness in response to the breakup of a particular relationship, this last factor being a more mature way of coping after the loss of a significant other. The self-criticism factor refers to a self-oriented variable and includes items regarding feelings of guilt, emptiness, hopelessness, dissatisfaction with oneself, failure to meet one’s own expectations, and the tendency to be critical about oneself. The questions are to be answered following a Likert-type scale ranging from 1 to 7, where 1 represents “strongly disagree” and 7 indicates “strongly agree.” Scores are calculated independently for each subscale since dependence and self-criticism are differentially associated with different types of depressive symptoms and interpersonal behaviors [30]. The minimum and maximum score on each subscale is 1 and 7, respectively. Although there is no cutoff point in the interpretation of this scale, higher scores indicate higher levels of self-criticism or dependence. In this version of the questionnaire, Cronbach α indicated good internal consistency for the 3 factors of dependence (α=0.82), self-criticism (α=0.85), and relationship (α=0.71).

Concerning the BSQ [31] we used the short version (BSQ-14) validated by Dowson and Henderson [32], which consists of 14 items measuring body dissatisfaction in the last 2 weeks using a 6-point Likert scale, where 1 is equivalent to “never” and 6 is to “always.” The total score is obtained by adding the score of each item so that it ranges from 14 to 84. Although there is no established cutoff point for body image dissatisfaction, higher scores indicate greater dissatisfaction or concern about body shape [33]. The Cronbach α of this version is 0.93, showing good internal reliability. In a study in which 8 different versions of this scale were compared, this was 1 of the 3 versions that showed the most favorable results in comparison to the original 34-item scale [34]. In a study with Mexican university women, an adequate fit (comparative fit index=0.95; normed fit index=0.92; goodness-of-fit index=0.86) and high internal consistency (α=0.96) were obtained [35]. In addition, it correlated positively with the Eating Attitudes Test-26, which measures the presence of eating disorder symptoms [36].

### Ethical Considerations

This study received approval (MPGS_2021_012) from the University of Comillas Research Ethics Committee and was compliant with the research ethics principles of the Declaration of Helsinki. An informed consent was obtained from every participant after acknowledging the nature and objectives of this study. This study’s data are anonymous and voluntary, as no compensation was offered to the participants.

### Statistical Analysis

Data analysis was conducted using SPSS Statistics (version 26; IBM Corp). For descriptive analysis of the data, qualitative variables are presented with a distribution of frequencies, and quantitative variables are presented using mean and SD. The continuous nonnormally distributed variables were summarized by the median (IQR 25th percentile to 75th percentile). To compare the groups, the ANOVA test for scores obtained on the scales and quantitative normally distributed variables and the nonparametric Kruskal-Wallis test for nonnormally distributed variables were used. Bonferroni test for multiple comparisons was applied to determine which means differed from each other. In the case of qualitative variables, comparison was evaluated using the chi-square test, or by the Fisher exact test in case more than 25% of the expected values were less than five. These analyses were performed to compare the 3 different Instagram usage times groups (less than 1 hour, between 1 and 3 hours, and more than 3 hours) and to compare scales of the most viewed content on Instagram. Subsequently, linear regression models were performed to explore the association between each of the dependent variables (SCS, self-criticism and dependency subscale of the DEQ, and the BSQ) and the time spent using Instagram daily and the type of content most viewed on this platform. The variables associated with the 3 different Instagram usage times groups (those variables which, in the univariate analyses, showed a level of statistical significance of *P*<.05) were considered to adjust the linear regression models. In the adjusted models, it was found that the effect of the Instagram usage times groups on the scales did not change by more than 15%. Therefore, the main model is considered without adjustment. Further, linear regression models were carried out with the total sample and by age groups (aged 18-35 and 35-50 years). For all the aforementioned tests, a significance value of 5% was accepted. Missing data due to nonresponse were not considered in the analyses. Therefore, for each variable, the analysis was of complete cases.

## Results

### Sociodemographic and Instagram Usage Characteristics of Participants

The final sample consisted of 1051 participants, from which 90.2% (948/1051) were women, 9% (95/1051) were men, 0.005% (5/1051) did not specify their gender, and 0.002% (2/1051) would rather not tell; mean age 30.58, SD 8.9 years). The sociodemographic data of our sample is presented in Table 1.

Descriptive data on participants’ Instagram usage is presented in Table 2. The vast majority of the sample (648/1051, 61.7%) use Instagram between 1 and 3 hours per day, compared to 25.2% (265/1051) and 13.1% (138/1051) of the participants who use it less than 1 hour per day and for more than 3 hours per day, respectively. A decrease in daily Instagram usage time was observed as age increased. Thus, 16% (63/394) of the group aged between 18 and 25 years use Instagram for more than 3 hours a day, while 15.1% (54/357) and 7% (21/300) of the participants in the groups of those aged 25-35 and 35-50 years, respectively, use Instagram for more than 3 hours a day.

**Table 1:** Sociodemographic variables^a^.

Variables		Total (N=1051)	Women (n=948)	Men (n=95)
Age (years), mean (SD)		30.58 (8.9)	30.77 (8.9)	24.23 (9.1)
**Age groups (years), n (%)**				
	18-25	394 (37.5)	344 (36.3)	44 (46.3)
	25-35	357 (34)	326 (34.4)	30 (31.6)
	35-50	300 (28.5)	278 (29.3)	21 (22.1)
**Educational level, n (%)**				
	Elementary education	1 (0.1)	1 (0.01)	0 (0)
	Mandatory high school education	40 (3.8)	35 (3.7)	4 (4.2)
	High school	162 (15.4)	141 (14.9)	18 (18.9)
	Professional study	187 (17.8)	163 (17.2)	22 (23.2)
	College degree	357 (34)	331 (34.9)	25 (26.3)
	Master’s degree	284 (27)	260 (27.4)	23 (24.2)
	Doctorate degree	20 (1.9)	17 (1.8)	3 (3.2)
**Work, n (%)**				
	Yes	688 (66.5)	627 (66.1)	58 (61.1)
	No	341 (32.4)	301 (31.8)	35 (36.8)
	Other	22 (2.1)	20 (2.1)	2 (2.1)
**Marital status, n (%)**				
	Single	371 (35.3)	310 (32.7)	55 (57.9)
	In a relationship	435 (41.4)	410 (43.2)	23 (24.2)
	Married	204 (19.4)	190 (20)	14 (14.7)
	Separated or divorced	35 (3.3)	32 (3.4)	3 (3.2)
	Other	6 (0.6)	6 (0.6)	0 (0)
**Children (yes), n (%)**		221 (21)	206 (21.7)	15 (15.8)
	Physical activity (yes)	616 (58.6)	550 (58)	64 (67.4)
	Substance use (yes)	546 (52)	480 (50.6)	64 (67.4)

^a^Sociodemographic characteristics of the total sample of participants and by sex. The variable “children (yes)” refers to participants having one or more children, “physical activity (yes)” refers to participants who practice some type of physical activity, and “substance use (yes)” refers to participants who consume some type of substance (alcohol, tobacco, or other).

**Table 2:** Descriptive data on Instagram usage of the total sample and by age group.

Variables		Total (N=1051)	Aged 18-25 years (n=394)	Aged 25-35 years (n=357)	Aged 35-50 years (n=300)
**Daily usage time^a^ (hours), n (%)**					
	<1	265 (25.2)	90 (23)	88 (24.6)	87 (29)
	1-3	648 (61.7)	241 (61.2)	215 (60.2)	192 (64)
	>3	138 (13.1)	63 (16)	54 (15.1)	21 (7)
**Followers^b^, n (%)**					
	<100 followers	275 (26.2)	36 (9.1)	86 (24.1)	153 (51)
	101-500 followers	530 (50.4)	205 (52)	207 (58)	118 (39.3)
	>500 followers	233 (22.2)	149 (37.8)	61 (17.1)	23 (7.7)
**Users followed^b^, n (%)**					
	<100	102 (9.7)	8 (2)	29 (8.1)	65 (21.7)
	101-500	577 (54.9)	203 (51.5)	205 (57.4)	169 (56.3)
	>500	359 (34.2)	181 (45.9)	119 (33.3)	59 (19.7)
**Most viewed content^c^, n (%)**					
	Sports, fitness, or lifestyle	96 (12.7)	33 (8.4)	35 (9.8)	28 (9.3)
	Beauty or fashion	134 (12.7)	45 (11.4)	51 (14.3)	38 (12.7)
	Family or friends	259 (24.6)	133 (33.8)	88 (24.6)	38 (12.7)
	Science	245 (23.3)	43 (10.9)	74 (20.7)	128 (42.7)
	Gastronomy	54 (5.1)	22 (5.6)	15 (4.2)	17 (5.7)
	Humor	146 (13.9)	75 (19)	51 (14.3)	20 (6.7)
	Travel	33 (3.1)	15 (3.8)	12 (3.4)	6 (2)
	News	17 (1.6)	3 (0.8)	6 (1.7)	8 (2.7)
	Other	67 (6.4)	25 (6.3)	25 (7)	17 (5.7)
**Time using Instagram^d^ (years), n (%)**					
	<4	197 (18.7)	35 (8.9)	58 (16.2)	104 (34.7)
	5-9	352 (33.5)	193 (49)	104 (29.1)	55 (18.3)
	>9	159 (15.1)	71 (18)	67 (18.8)	21 (7)

^a^Daily usage time: expressed on several minutes or hours on app usage time settings.

^b^Followers and users you follow: expressed by directly observing Instagram account statistics.

^c^Most viewed content: participants expressed their 3 most observed content, in order from highest to lowest consumption.

^d^Time using Instagram: expressed by observing the year they created their Instagram account.

### Correlation Between the Most Observed Type of Content and Psychological Variables

The total sample obtained an average of 2.75 (SD 0.41) on the SCS, an average of 3.61 (SD 1.76) on the self-criticism subscale of the DEQ (DEQ-A), 3.73 (SD 1.58) on the dependence subscale of the DEQ (DEQ-D) and, finally, an average of 47.69 (SD 20.10) on the BSQ-14. There are significant statistical differences between the 3 age groups in the scores obtained in both subscales of the DEQ (*P*<.001), in BSQ-14 (*P*<.001), and in the SCS scale (*P*=.005).

The Bonferroni test indicated that in the 3 scales used in our investigation, there were significant differences between the scores of the youngest group (aged 18-25 years) and the oldest group (35-50 years), but not between the scores of the age groups of 18-25 years and 25-35 years. Due to this, the younger and middle-aged groups were combined to create a single group compromising participants aged 18-35 years. The results displayed in Table 3 refer to 2 age groups, one including participants aged 18-35 years and the other including those aged 35-50 years. The *t* test (2-tailed) for independent samples showed that there are significant statistical differences in the scores obtained in the 3 scales between the aged 18-35 years group and the aged 35-50 years group.

**Table 3:** Descriptive data for the total sample and scales mean comparisons between different age groups.

Dependent variables	Total	Aged 18-35 years	Aged 35-50 years	*P* value^a^
SCS^b^, mean (SD)	2.75 (0.41)	2.73 (0.41)	2.81 (0.41)	.002
DEQ-A^c^, mean (SD)	3.61 (1.76)	3.80 (1.74)	3.13 (1.7)	<.001
DEQ-D^d^, mean (SD)	3.73 (1.58)	3.82 (1.63)	3.49 (1.4)	.002
BSQ^e^, mean (SD)	47.69 (20.1)	49.23 (20.43)	43.83 (18.73)	<.001

^a^*P*<.05.

^b^SCS: Self-Compassion Scale.

^c^DEQ-A: self-criticism subscale of the Depressive Experiences Questionnaire.

^d^DEQ-D: dependency subscale of the Depressive Experiences Questionnaire.

^e^BSQ: Body Shape Questionnaire.

### Relation Between Time Using Instagram and Self-Criticism

Mean scores and SDs of the scales as a result of time spent using Instagram, as well as the *P* value after performing ANOVA analysis are presented in Table 4. The results revealed significant statistical differences across the 3 different usage times groups in the mean scores obtained in the DEQ-A and DEQ-D (*P*<.001). However, no significant statistical differences were found in the mean scores obtained in the SCS (*P*=.12) or the BSQ-14 (*P*=.43) among the 3 different times of use.

**Table 4:** Scores of the scales and mean comparison according to daily Instagram time of use.

Dependent variables	Less than 1 hour	Between 1 and 3 hours	More than 3 hours	*P* value
SCS^a^, mean (SD)	2.79 (0.39)	2.76 (0.41)	2.69 (0.42)	.12
DEQ-A^b^, mean (SD)	3.30 (1.69)	3.63 (1.72)	4.12 (1.94)	<.001
DEQ-D^c^, mean (SD)	3.53 (1.55)	3.71 (1.55)	4.21 (1.64)	<.001
BSQ-14^d^, mean (SD)	46.38 (19.75)	48 (20.05)	48.75 (21.03)	.43

^a^SCS: Self-Compassion Scale.

^b^DEQ-A: self-criticism subscale of the Depressive Experiences Questionnaire.

^c^DEQ-D: dependency subscale of the Depressive Experiences Questionnaire.

^d^BSQ-14: Body Shape Questionnaire.

### Type of Content Viewed Is Related to Body Dissatisfaction

Descriptive data of the scales according to the type of content most viewed on Instagram and the results of the 1-factor ANOVA analysis are presented in Table 5. In such analysis, the 5 types of content most viewed by the participants (family or friends, science, humor, beauty or fashion, and sport, fitness, or lifestyle) were analyzed. Statistically significant differences were found only in body dissatisfaction (*P*=.02) according to the type of most viewed content. However, no statistically significant differences were observed in self-compassion, self-criticism, and dependence when taking the most observed content type into account (*P*=.36, *P*=.16, and *P*=.07, respectively). Bonferroni test results indicated that there are statistically significant differences in the mean scores of body dissatisfaction between those groups whose most observed content was “science” and “humor” (*P*=.007).

Results of the linear regression model conducted to explore the relationships between daily Instagram use and self-compassion, self-criticism, and body dissatisfaction are presented in Table 6. To begin with, no statistically significant relationship was found between using Instagram between 1 and 3 hours per day or for more than 3 hours per day and SCS. As for self-criticism (DEQ-A), a statistically significant association was observed between this variable and using Instagram between 1 and 3 hours a day (*P*=.01) and for more than 3 hours a day (*P*<.001). Participants who use Instagram between 1 and 3 hours a day scored on average 0.325 (SD 0.13) points higher on this subscale than those who use Instagram for less than 1 hour a day. On the other hand, those who use Instagram for more than 3 hours a day scored on average 0.817 (SD 0.18) points higher on this subscale than those who use it for less than 1 hour a day. As for dependence (DEQ-D), a statistically significant relationship was observed between this variable and using Instagram for more than 3 hours a day (*P*<.001), but not between using Instagram between 1 and 3 hours a day (*P*=.11). Compared to those who use Instagram less than one hour a day, those who use this social network for more than 3 hours a day scored on average 0.680 (SD 0.16) points higher on this subscale. Finally, no statistically significant relationship was found between consuming Instagram between 1 and 3 hours a day or for more than 3 hours a day on body dissatisfaction.

**Table 5:** Descriptive data and mean comparison on the self-compassion, self-criticism, and body dissatisfaction scales and the types of content most frequently observed by the participants.

DV^a^	Sport, fitness, or lifestyle	Beauty or fashion	Family or friends	Science	Humor	*P* value
SCS^b^	2.76 (0.39)	2.73 (0.37)	2.75 (0.42)	2.80 (0.42)	2.70 (0.39)	.36
DEQ-A^c^	3.47 (1.7)	3.85 (1.77)	3.56 (1.69)	3.48 (1.72)	3.76 (1.76)	.16
DEQ-D^d^	3.49 (1.66)	3.92 (1.58)	3.81 (1.54)	3.57 (1.49)	3.74 (1.6)	.07
BSQ-14^e^	50.06 (21.19)	47.38 (18.7)	48.12 (20.2)	43.96 (19.49)	51.82 (21.37)	.02

^a^DV: dependent variables.

^b^SCS: Self-Compassion Scale.

^c^DEQ-A: self-criticism subscale of the Depressive Experiences Questionnaire.

^d^DEQ-D: dependency subscale of the Depressive Experiences Questionnaire.

^e^BSQ-14: Body Shape Questionnaire.

**Table 6:** Unadjusted linear regression model and *P* value for the association between psychological variables and daily Instagram use in the overall sample (N=1051)^a^.

	SCS^b^		DEQ-A^c^			DEQ-D^d^			BSQ-14^e^	
	β^f^	*P* value	β	*P* value	β		*P* value	β		*P* value
Between 1 and 3 hours	–0.11	.71	0.325	.01	0.184		.11	1.624		.27
More than 3 hours	–0.83	.05	0.817	<.001	0.680		<.001	2.376		.26

^a^Reference category in a linear regression model to which other times of usage were compared to less than 1 hour.

^b^SCS: Self-Compassion Scale.

^c^DEQ-A: self-criticism subscale of the Depressive Experiences Questionnaire.

^d^DEQ-D: dependence subscale of the Depressive Experiences Questionnaire.

^e^BSQ-14: Body Shape Questionnaire.

^f^β: regression coefficient.

### Age Differences Between Daily Instagram Use and Psychological Variables

Results of the linear regression model conducted to explore the relationships between daily Instagram usage time and self-compassion, self-criticism, dependence, and body dissatisfaction by age group are shown in Table 7. No statistically significant relationships were found between using Instagram between 1 and 3 hours per day and for more than 3 hours per day and self-compassion and body dissatisfaction scores were not found in either of the 2 age groups. However, in both groups, a statistically significant relationship was observed between using Instagram for more than 3 hours a day and self-criticism. In the aged 18-35 years group, those using Instagram for more than 3 hours a day scored on average 0.615 (SD 0.21) points higher on the self-criticism subscale than those using Instagram for less than 1 hour daily (*P*=.003). In the aged 35- to 50-year group, people who use Instagram for more than 3 hours a day scored on average 1.111 (SD 0.41) points higher on this subscale than people who use Instagram for less than 1 hour per day (*P*=.007). Regarding the dependence variable, a statistically significant relationship was observed between consuming Instagram for more than 3 hours a day in both age groups, but not between using Instagram 1-3 hours a day and this variable. In this sense, individuals aged between 18 and 35 years who consume Instagram for more than 3 hours a day scored on average 0.571 (SD 0.19) points higher in dependency than those who use this social network for less than 1 hour a day (*P*=.003). Regarding the 35- to 50-year age group, individuals who use Instagram for more than 3 hours a day scored on average 0.911 (SD 0.34) points higher in dependence than those who use Instagram for less than 1 hour (*P*=.007).

**Table 7:** Unadjusted linear regression estimate and *P* value for the association between psychological variables and daily Instagram use stratified by age^a^.

		SCS^b^		DEQ-A^c^			DEQ-D^d^			BSQ-14^e^	
		β^f^	*P* value	β	*P* value	β		*P* value	β		*P* value
**Aged 18-35 years (n=751)**											
	Between 1 and 3 hours	–0.019	.59	0.277	.07	0.165		.25	0.969		.59
	More than 3 hours	–0.075	.12	0.615	.003	0.571		.003	0.035		.99
**Aged 35-50 years (n=300)**											
	Between 1 and 3 hours	0.015	.78	0.362	.097	0.193		.28	2.435		.32
	More than 3 hours	–0.062	.54	1.111	.007	0.911		.007	7.443		.10

^a^Reference category in a linear regression model to which other times of usage were compared to less than 1 hour.

^b^SCS: Self-Compassion Scale.

^c^DEQ-A: self-criticism subscale of the Depressive Experiences Questionnaire.

^d^DEQ-D: dependence subscale of the Depressive Experiences Questionnaire.

^e^BSQ-14: Body Shape Questionnaire.

^f^β: regression coefficient.

**Psychological Variables Affected by the Most Observed Type of Content**

Table 8 provides the results of the linear regression model that analyzed the relationship between the type of most viewed content on Instagram and self-compassion, self-criticism, dependence, and body dissatisfaction. Regarding the self-compassion variable, the linear regression equation was statistically significant only for the “humor” content type (*P*=.02). Participants who mainly watch “humor” content scored on average 0.100 (SD 0.04) points lower in self-compassion than those who watch mostly science. Regarding self-criticism, no statistically significant relationship was found between this variable and the type of most watched content on Instagram. Regarding dependence, only a statistically significant relationship was found between this variable and watching mostly “beauty or fashion” content (*P*=.04). Compared to individuals whose most viewed content on Instagram is “science,” individuals who view mainly “beauty or fashion” content scored on average 0.355 (SD 0.17) points higher in dependence. Finally, a statistically significant relationship was observed between body dissatisfaction and observing mainly “sport, fitness, or lifestyle” content (*P*=.01), “family or friends” (*P*=.02), and “humor” (*P*<.001). Individuals who mainly view “sports, fitness, or lifestyle” content scored on average 6.099 (SD 2.41) points higher in body dissatisfaction than those whose most viewed content is “science.” On the other hand, individuals whose most viewed content is “family or friends” and “humor” scored on average 4.156 (SD 1.78) and 7.859 (SD 2.10) higher in body dissatisfaction, respectively, than those who mainly watch “science” content. However, no statistically significant association was found between watching mostly “beauty or fashion” content and body dissatisfaction.

**Table 8:** Unadjusted linear regression model and *P* value for the association between psychological variables and the most observed type of content in the overall sample^a^.

	SCS^b^		DEQ-A^c^			DEQ-D^d^			BSQ-14^e^	
Most viewed content	β^f^	*P* value	β	*P* value	β		*P* value	β		*P* value
Sports, fitness, or lifestyle	–0.041	.40	–0.002	.99	–0.085		.66	6.099		.01
Beauty or fashion	–0.068	.12	0.366	.05	0.355		.04	3.417		.11
Family or friends	–0.045	.22	0.078	.62	0.240		.09	4.156		.02
Humor	–0.100	.02	0.268	.16	0.170		.30	7.859		<.001

^a^Reference category in a linear regression model to which other types of content were compared to science.

^b^SCS: Self-Compassion Scale.

^c^DEQ-A: self-criticism subscale of the Depressive Experiences Questionnaire.

^d^DEQ-D: dependence subscale of the Depressive Experiences Questionnaire.

^e^BSQ-14: Body Shape Questionnaire.

^f^β: regression coefficient.

### Impact of the Most Viewed Type of Content on Younger Individuals

At last, Table 9 displays results from the linear regression model that analyzed the relationship between the type of most viewed content on Instagram and self-compassion, self-criticism, dependence, and body dissatisfaction by age group. No statistically significant relationship was found in any of the age groups between the type of most viewed content on Instagram and self-compassion. In regards to the aged 18-35 years group, a statistically significant relationship was observed between self-criticism and watching mostly “beauty or fashion” content (*P*=.03). Individuals in this age group who watch mainly “beauty or fashion” content scored on average 0.530 (SD 0.24) points higher in self-criticism than those who mainly watch “science” content. On the other hand, a statistically significant relationship was observed between dependence and mainly watching “beauty or fashion” content (*P*=.02). Compared to individuals whose most watched content is “science,” individuals who mainly watch “beauty or fashion” content scored on average 0.508 (SD 0.22) points higher in dependence. As for body dissatisfaction, a statistically significant relationship was observed only between this variable and observing mainly “humor” content (*P*=.01). Individuals aged 18-35 years who consume mainly “humor” content scored on average 6.46 (SD 2.62) points higher in body dissatisfaction than those who consume mainly “science” content. In the aged 35-50 years group, no statistically significant relationships were observed between the type of content most viewed on Instagram and the studied variables.

**Table 9:** Unadjusted linear regression model and *P* value for the association between psychological variables and the most observed type of content stratified by age^a^.

		SCS^b^		DEQ-A^c^		DEQ-D^d^		BSQ-14^e^	
		β^f^	*P* value	β	*P* value	β	*P* value	β	*P* value
**Aged 18-35 years (n=751)**									
	Sports, fitness, or lifestyle	–0.013	.84	0.059	.82	0.015	.95	5.212	.09
	Beauty or fashion	–0.070	.21	0.530	.03	0.508	.02	3.044	.28
	Family or friends	–0.005	.91	–0.017	.93	0.273	.14	2.865	.22
	Humor	–0.071	.17	0.096	.67	0.207	.32	6.460	.01
**Aged 35-50 years (n=300)**									
	Sports, fitness, or lifestyle	–0.049	.56	–0.453	.20	–0.351	.23	5.156	.19
	Beauty or fashion	0.005	.95	–0.370	.24	–0.055	.83	1.064	.76
	Family or friends	–0.136	.08	–0.346	.27	–0.025	.93	1.643	.64
	Humor	–0.051	.60	0.214	.60	–0.146	.67	5.656	.21

^a^Reference category in a linear regression model to which other types of content were compared to science.

^b^SCS: Self-Compassion Scale.

^c^DEQ-A: self-criticism subscale of the Depressive Experiences Questionnaire.

^d^DEQ-D: dependence subscale of the Depressive Experiences Questionnaire.

^e^BSQ-14: Body Shape Questionnaire.

^f^β: regression coefficient.

## Discussion

### Principal Findings

We investigated the impact of Spanish people’s Instagram use on psychological well-being as assessed through the domains of self-compassion, self-criticism, and body dissatisfaction. The innovations in our study include an assessment of the type of content and the total time of use, together with the impact on these psychological domains. According to our findings, greater Instagram daily time of use is associated with higher self-criticism scores in participants of all ages. In regards to the most viewed type of content on this social network, observing primarily sports or fitness and family or friends content predicted higher body dissatisfaction scores in all the samples. At last, observing predominantly beauty or fashion content predicted higher self-criticism scores only among the youngest participants.

There was a positive association between Instagram usage (we refer to duration of use as usage hereinafter) and self-criticism. Users who spent more time on Instagram, particularly those spending over 3 hours per day on the platform, exhibited higher self-criticism scores. When we analyzed self-criticism to the time spent on Instagram within different age groups, we found a positive and significant association between consuming Instagram for more than 3 hours a day in both age groups, but no association with using the social network for 1-3 hours a day. These findings align with previous research exploring the link between Instagram usage time and other variables related to self-criticism. In a recent study, greater Instagram usage time was associated with higher levels of concern and yearning for perfection [8]. Additionally, problematic use of Facebook and Instagram (characterized by a strong motivation to access the social network and loss of control over usage time impacting psychological well-being) has been linked to negative self-oriented perfectionism and socially prescriptive perfectionism [37].

However, we did not find a statistically significant relationship between Instagram usage time and self-compassion in either of the 2 age groups. In contrast to our findings, a different study found that higher Instagram use was associated with lower levels of self-compassion and poorer psychological well-being [13]. In that same study, individuals with higher levels of self-compassion tended to spend less time using Instagram.

No association was found between body dissatisfaction and daily Instagram usage. These unexpected findings contrast with previous studies that indicated greater daily usage of Instagram was associated with a greater tendency to think about one’s appearance, body dissatisfaction, and lower self-esteem and psychological well-being [7,10,12,38]. The results from a recent study showed that the relationship between Instagram use and body image concerns is mediated by comparisons related to physical appearance [7]. Women who engage in appearance-related comparisons on Instagram and Facebook are more vulnerable to body dissatisfaction, particularly regarding their face, skin, and hair [39,40]. Social interactions on Facebook, such as looking at other profiles, liking, and leaving comments, have also been associated with higher levels of body dissatisfaction, but this variable was not associated with overall Facebook exposure [41]. These findings suggest that user body dissatisfaction may be influenced not just by the duration of Instagram use, but also by the nature of social comparisons and interactions regarding physical appearance. It is possible that duration of use does not fully explain the variation in users’ body dissatisfaction, and other variables may be more relevant in explaining the relationship between Instagram usage and body dissatisfaction.

We found age differences for the impact of the type of content viewed. For those aged 18-35 years, a positive association was observed between self-criticism and predominantly viewing “beauty or fashion” content. Compared to those aged 18-35 years who primarily observe “science” content, those who mainly watch “beauty or fashion” content had higher self-criticism scores. It has been found that Instagram users regularly post pictures about their physical appearance that often feature filters or have been previously edited [7], promoting beauty standards and stereotypes that are difficult to achieve for most people [5,42,43]. Viewing images based on stereotyped beauty and idealized body forms increases the likelihood that users of this social network will engage in upward social comparison, leading to feelings of inferiority [42,44]. Those who predominantly observe “beauty or fashion” content may have a greater tendency to be more self-critical and experience greater feelings of inferiority, as they perceive the people depicted in such images to be more attractive than themselves.

However, in those aged 35-50 years, we found no correlation between predominantly viewing “beauty or fashion” content and self-criticism. It is plausible that participants aged 35-50 years may be less likely to compare themselves to the Instagram “beauty or fashion” images and might then have more realistic expectations about their self-image, which could decrease the likelihood of experiencing feelings of inferiority and the development of a negative view of themselves. Older women are less affected by beauty and thinness stereotypes promoted by society and, therefore, have higher self-esteem and psychological well-being [45-48]. Moreover, older people set more realistic expectations about themselves and are less concerned about their social status [49,50].

There was a significant association between body dissatisfaction and predominantly observing “sport, fitness, or lifestyle,” “family or friends,” and “humor” content. These findings are consistent with previous research where observing “fitness” images has been linked to lower body satisfaction and a greater desire to achieve thinness [40,51,52]. The internalization of beauty standards and the tendency to compare one’s own appearance with that of women in fitness images likely mediates the development of body image concerns [40]. A recent study found that comparing oneself to idealized bodies perceived as more attractive led to greater weight and appearance dissatisfaction and lower confidence [53]. In this study, women who were more perfectionistic about their appearance experienced greater weight and appearance dissatisfaction after comparing themselves to Instagram models, compared to women who did not worry about having an imperfect appearance. Furthermore, the positive relationship between predominantly observing “family or friends” content and body dissatisfaction may also be explained by the phenomenon of upward social comparison. Family members and friends may use Instagram’s filters and image editing strategies, presenting a version of themselves that does not correspond to their true self, but rather an attempt to achieve beauty stereotypes frequently promoted on this platform. Therefore, observing this type of content may lead users to perceive their physical appearance as less attractive than that of their relatives or friends.

However, there was no significant association between the most viewed content type and self-compassion, except “humor” content, which had a negative association with self-compassion. This contrasts with previous research finding women and men who viewed physical appearance-related content (images of muscular and toned bodies) reported lower levels of self-compassion compared to those who viewed neutral images about architecture [51,52]. The lack of our predicted positive relationship between predominantly observing appearance-related content and self-compassion may be explained by the emergence of health campaigns in fashion and social media, such as the “body positive” movement, that challenges dominant beauty and idealized body stereotypes as well as promoting acceptance and appreciation of all body types and self-compassion [52,54,55]. The lack of a statistically significant relationship between predominantly consuming beauty or fashion content and body dissatisfaction may be elucidated by the potential positive influence of the aforementioned campaigns. Furthermore, participants in our study may perceive beauty or fashion content as emphasizing characteristics related to their face, makeup, hair, or attire, in contrast to the sports, fitness, or lifestyle category, which may be perceived as centered on body shape and health. The instrument used to measure body dissatisfaction in our study specifically targeted aspects related to body shape, rather than encompassing characteristics of other anatomical regions, such as the face. This specificity in measurement focus could contribute to the observed absence of a significant relationship between body dissatisfaction and beauty or fashion content.

We found higher Instagram usage time is associated with higher levels of self-criticism and that viewing physical appearance-related content on Instagram, such as beauty or fashion content and fitness, sports, or lifestyle content is associated with higher levels of self-criticism in the youngest participants and body dissatisfaction in the general sample, respectively. Although our study did not find an association between Instagram use and self-compassion, perhaps interventions that target the development of a self-compassionate attitude may contribute to a more positive vision that users have of themselves compared to others. Self-compassion may have a protective function against stressful situations in both adults and adolescents [23]. A nonjudgmental, accepting attitude toward personal characteristics and body image might help decrease the impact of Instagram-related social comparisons and self-criticism, and promote greater psychological well-being.

Related research on general social media use, such as the viewing of news during disasters, has demonstrated negative impacts on psychological well-being associated with duration of use. For example, social media use of more than or equal to 2 hours per day was associated with posttraumatic stress disorder and depressive symptoms in a longitudinal study of social unrest in Hong Kong from 2009 to 2019 [56]. Similarly, a systematic review of social networking site use found that differing engagement styles might explain the development of anxiety or depression, as we found for Instagram users in our study [57]. However, this broader systematic review did not identify a particular usage time that predicted effects. There is related literature on problematic social media usage, such as the use of Facebook, where users display addictive-type behavior that is often associated with the development of anxiety and depression [58].

### Limitations

However, our study has some limitations that may affect the interpretation of our results. First, due to the cross-sectional nature of this study, causality cannot be attributed to the observed associations. Second, we have not conducted a clinical evaluation or a comprehensive psychological characterization of the participants beyond the use of self-administered questionnaires and scales. Conducting such evaluations would have added value to this study, considering that certain personality traits have been associated with a predisposition to internalize ideals propagated through media [59]. Third, we did not control for the absence of depression or anxiety diagnoses among our participants, which could have potentially influenced our results. Higher scores of self-criticism and body dissatisfaction may be indicative of symptoms related to these disorders rather than a direct consequence of Instagram use. Fourth, the majority of participants in our study were women, which limits our ability to assess the influence of gender on the results. We also could not evaluate the impact of culture since all participants were of Spanish nationality. Therefore, we do not know if our results are generalizable to individuals from other cultures.

### Conclusions

Increased Instagram usage, especially for more than 3 hours per day, may hurt individuals’ self-perception and beliefs concerning their physical appearance, and, through increased self-criticism reduce psychological well-being. Perhaps it is time to develop evidence-based guidelines on how to adopt a balanced approach to using social media, particularly visually oriented platforms such as Instagram. Further longitudinal studies to examine the effects of usage of Instagram and other social media platforms on body image and self-perception are needed to inform the development of more detailed evidence-based usage guidelines. In the interim, based on our research, perhaps Instagram users focusing on physical appearance content should limit their use to less than three hours a day to avoid negative effects on psychological well-being from social comparison.

Furthermore, these guidelines should take into account that the psychological impact of social media usage varies according to age, with younger individuals being more susceptible. Future longitudinal studies that examine the relationship between the type of Instagram usage and other social media platforms according to gender and culture are needed to provide a more exhaustive comprehension of these platforms and elaborate more specific interventions and guidelines on social media usage.

## Appendix

Multimedia Appendix 1 Questionnaire on Instagram usage.

## Abbreviations

BSQ-14: Body Shape Questionnaire
DEQ: Depressive Experiences Questionnaire
SCS: Self-Compassion Scale

## Data Availability

The data sets generated during and analyzed during this study are available from the corresponding author upon request.
