# Supplementary material for: Exploring the Relationship Between Instagram Use and Self-Criticism, Self-Compassion, and Body Dissatisfaction in the Spanish Population: Observational Study
Source: J Med Internet Res. 2024 Aug 1;26:e51957. doi: 10.2196/51957 (PMC11327623; doi:10.2196/51957)
Supplement: Multimedia Appendix 1 [file jmir_v26i1e51957_app1.pdf]

Questionnaire on Instagram usage

1. What social media platforms do you use? Indicate all the ones that you use.
   - Facebook.
   - Instagram.
   - Twitter.
   - Snapchat.
   - TikTok.
   - Others: ____________

2. Select the social media platform you use the most:
   - Facebook.
   - Instagram.
   - Twitter.
   - Snapchat.
   - TikTok.
   - Other: ____

3. For how long (years or months) have you been using Instagram?

4. How much time do you use Instagram per day? (To answer this question, please check the exact time as follows: iPhone: Settings > Screen Time > See All Activity > Look at the time under "Instagram"; Android: Settings > Digital Wellbeing & Parental Controls > Tap on the number of minutes you have used your phone today > Look at the time under "Instagram").

   A) Less than 1 hour.
   B) 1-3 hours per day
   C) More than 3 hours per day

5. Do you use Instagram when you are feeling bored?
   A) Always
   B) Sometimes
   C) Rarely
   D) Never

6. How many people do you follow on Instagram? _______

7. How many people follow you on Instagram? _______

8. What kinds of accounts do you follow on Instagram? Choose the three types of accounts you follow the most.
   a) Science (psychology, medicine, nutrition, etc.).

b) Gastronomy
c) Travel
d) Family and friends
e) Beauty/Fashion
f) Sports/Fitness/Lifestyle
g) Humor
h) News
i) Others: ____

9. Select the three types of content you typically view on Instagram.
   a) Science (psychology, medicine, nutrition, etc.).
   b) Gastronomy
   c) Travel
   d) Family and friends
   e) Beauty/Fashion
   f) Sports/Fitness/Lifestyle
   g) Humor
   h) News
   i) Others: ____

10. Rank the three types of content you selected in the previous question from the most to least watched, with 1 being the most observed and 3 the least observed.
    (1) ______________
    (2) ______________
    (3) ______________

11. Indicate the percentage of time you spend viewing each type of content you selected in the previous question, making sure the total adds up to 100%. For example, if you use Instagram for 3 hours a day, you might spend 50% of the time on humor content, 30% on science content, and 20% on fitness content.
    - Percentage of time dedicated to viewing content type (1) specified in the previous question: ______
    - Percentage of time dedicated to viewing content type (2) specified in the previous question: ______
    - Percentage of time dedicated to viewing content type (3) specified in the previous question: ______

12. How many posts do you typically upload to Instagram PER WEEK? (This refers to posts on your profile feed).
    a. 0
    b. 1-5
    c. More than 5

13. How important are the number of likes, followers, or comments you receive on Instagram to you?
    a. A lot
    b. Moderate

c. Little
d. None

14. How many stories do you typically upload to Instagram PER DAY?
   a. 0
   b. 1-5
   c. More than 5

15. Proportionate to your number of followers, how many likes and comments do you receive?
   a. Many
   b. Some
   c. Few
   d. None

16. Do you feel good about yourself when you post a photo of yourself and it gets a lot of likes or comments?
   a. Always
   b. Sometimes
   c. Rarely
   d. Never

17. Do you edit photos or use filters before posting them on Instagram?
   a. Always
   b. Sometimes
   c. Rarely
   d. Never

18. Do you feel bad about yourself when you post a photo of yourself and it doesn't get a lot of likes or comments?
   a. Always
   b. Sometimes
   c. Rarely
   d. Never

19. Do you delete your posts or stories if they don't get many likes or comments?
   a. Always
   b. Sometimes
   c. Rarely
   d. Never

20. Do you use Instagram for any economic purposes?
   - Yes
   - No

21. If you answered yes to the previous question, is Instagram your primary source of income or is it a supplementary income alongside your main job?
   - Main source of income.
   - Supplement to my main job.
